# Rotavirus infection and breastfeeding practices among children under two: Findings from a tertiary hospital in Chattogram, Bangladesh (2018-20)

**DOI:** 10.1016/j.ijregi.2025.100742

**Published:** 2025-09-03

**Authors:** Muhammad Gias Uddin, Immamul Muntasir, Mehejabin Nurunnahar, Mohammed Hossain, Mohammad Belal Uddin, Mohammad Toufiqul Islam, Samia Afser, Mohammad Rashedul Hassan, Nasir Uddin Mahmud, Md. Omar Qayum

**Affiliations:** 1 Bangladesh Institute of Tropical and Infectious Diseases (BITID), Ministry of Health & Family Welfare (MoHFW), Chattogram, Bangladesh; 2 Institute of Epidemiology, Disease Control and Research (IEDCR), Ministry of Health & Family Welfare (MoHFW), Dhaka, Bangladesh; 3 Upazila Health Complex, Ministry of Health & Family Welfare (MoHFW), Sitakunda, Chattogram, Bangladesh; 4 Department of Paediatric Neurology and Development, Chattagram Maa O Shishu Hospital Medical College, Chattogram, Bangladesh; 5 Upazila Health Complex, Ministry of Health & Family Welfare (MoHFW), Chandpur, Chattogram, Bangladesh; 6 Faculty of Biological Science, University of Chittagong, Chattogram, Bangladesh; 7 Institute of Public Health Nutrition (IPHN), Dhaka, Bangladesh

**Keywords:** Rotavirus infections, Breast feeding, Bangladesh, Odds ratio, Inpatients

## Abstract

**Objectives:**

To determine the prevalence of rotavirus infection and explore the association between exclusive breastfeeding (EBF) and rotavirus-related diarrhea among children under 2 years of age in Bangladesh.

**Methods:**

A cross-sectional observational study was conducted between July 2018 and June 2020 in the Pediatric Inpatient Department of Chittagong Medical College Hospital. A total of 226 children under 2 years hospitalized with acute watery diarrhea were enrolled. Stool samples were tested for rotavirus. Data on demographics, feeding practices, vaccination, and clinical features were collected. Associations were assessed using odds ratios and statistical tests.

**Results:**

Among the 226 children, 60% tested positive for rotavirus. Children aged 7-12 months had the highest infection rate (64%). EBF was significantly protective: in children ≤6 months, non-EBF was associated with a 10-fold higher risk of infection (odds ratio: 10.0; 95% confidence interval: 4.81-17.97; *P* =.04). For children >6 months, the risk was 9.72 times higher (*P* <.001). Nearly all children (98%) were unvaccinated.

**Conclusions:**

Rotavirus was highly prevalent among non-exclusively breastfed children. These findings emphasize the need to promote EBF and expand rotavirus vaccination coverage to reduce pediatric diarrheal morbidity.

## Highlights

• 60% of children hospitalized for diarrhea tested positive for rotavirus.
• Non-exclusively breastfed children had a 10-fold higher risk of rotavirus infection.
• Most infections occurred in the 7–12-month age group.
• Only 1.8% of children had received rotavirus vaccination.
• Exclusive breastfeeding showed strong protective effects against rotavirus.

## Introduction

Diarrheal diseases are a global public health problem and a leading cause of morbidity and mortality worldwide. According to the latest Global Burden of Disease Study, about 2.39 billion diarrheal cases occurred globally, and approximately 0.53 million children under 5 years of age died every year [1,2]. Specifically, incidence and case-fatality ratios are much higher in low- and middle-income countries [3]. In Bangladesh, diarrheal diseases are still very common among children under 5 years old [4].

Although diarrhea is preventable and can be managed with low-cost interventions, it remains the top cause of morbidity among patients seeking care in the public hospital system in Bangladesh, and significant resources are expended in treating these patients [5]. Considering the direct and indirect household costs, it also represents a substantial economic burden for affected families. A study conducted in six randomly selected district hospitals from six divisions in Bangladesh captured inpatient and outpatient treatment costs of diarrheal disease and measured the cost burden and coping mechanisms associated with diarrheal illness. The average total cost of illness per episode was 5274.02 taka, whereas the average inpatient and outpatient costs were 8675.09 taka and 1853.96 taka, respectively. The cost burden was significantly highest for the poorest households, 21.45% of household income, compared with 4.21% for the richest quintile [6].

Rotavirus, the most common cause of severe diarrhea in infants and young children, remains a significant cause of morbidity and mortality worldwide [7]. In 2010, approximately 200,000 global diarrhea deaths among children 5 years of age and younger were attributed to rotavirus, with about 25% occurring in Southeast Asia [7]. In Bangladesh, single-center studies have demonstrated a considerable burden of rotavirus on healthcare systems; rotavirus was the etiology of gastroenteritis in 20% of children seeking care at a diarrhea treatment facility in urban Dhaka, [8] and in 33% of hospitalizations for gastroenteritis in rural Matlab [9]. Rotavirus is a major cause of morbidity in Bangladeshi children, accounting for nearly two-thirds of acute gastroenteritis-related hospitalizations [10].

In anticipation of introducing a rotavirus vaccine into the national immunization program of Bangladesh, active hospital-based surveillance was initiated in seven tertiary hospitals located in seven divisions of Bangladesh to provide baseline pre-vaccine data on rotavirus diseases. Rotavirus was detected in 2432 (64%) of 3783 children 5 years of age and younger hospitalized for acute gastroenteritis from July 2012 to June 2015. Sixty-nine percent of children with rotavirus had severe disease (Vesikari score, ≥11). These data highlight the potential value of preventive programs against rotavirus diarrhea [10].

Prevention can be achieved by vaccination; however, not within the first 2 months of life. Breastfeeding is considered protective against infections. Epidemiological findings on the protective effect of breastfeeding, however, are inconsistent. Some studies did not show an effect of breastfeeding against rotavirus infection, while others found that breastfeeding may alleviate the severity of symptoms. Nevertheless, the majority of investigations reported a protective effect against rotavirus infections [11].

The global rate of exclusive breastfeeding (EBF) remains below 40% despite extensive efforts, including the implementation of the International Code of Marketing of Breast-Milk Substitutes, the Baby-Friendly Hospital Initiative, and the launching of the WHO/UNICEF Global Strategy for Infant and Young Child Feeding [12]. Progress has been similarly slow in South Asia. EBF increased only slightly from 40% in 1995 to 43% in 2009 (World Health Statistics, 2009). Based on 24-hour recall reported in the national Bangladesh Demographic and Health Surveys (BDHS) of 1999, 2004, and 2007, the proportion of EBF among infants younger than 6 months of age has not significantly increased in the past 12 years. BDHS 2007 reported 43%, rising to 64% in 2011 and then declining to 55% in 2014 [[13], [14], [15]].

On this background, we designed a study based on the assumption that breastfeeding is still being practiced in Bangladesh in accordance with cultural norms and recommendations from the WHO as an intervention to reduce the incidence and severity of diarrheal disease. However, its protective role against rotavirus is not well established. This study aimed to determine the proportion of rotavirus diarrhea among children aged less than 24 months of age hospitalized for diarrhea at Chittagong Medical College Hospital, to describe the pattern of breastfeeding practice, and to investigate the relationship between breastfeeding and rotavirus diarrhea in our setting.

## Methods

This hospital-based cross-sectional observational study was conducted over a 2-year period, from July 2018 to June 2020, in the Inpatient Department of Pediatrics at Chittagong Medical College Hospital (CMCH), Chattogram, Bangladesh. The study aimed to determine the association between EBF and rotavirus infection among hospitalized children under 2 years of age presenting with acute watery diarrhea.

### Study population and eligibility criteria

Children less than 24 months of age who were admitted with acute watery diarrhea (AWD) were eligible for inclusion. Acute watery diarrhea was defined as the passage of three or more watery or looser-than-normal stools, with or without at least one episode of forceful vomiting within a 24-hour period, with symptoms persisting for no more than 7 days. Children with bloody diarrhea, known immunodeficiencies, or incomplete clinical records were excluded.

### Sample size and sampling technique

The required sample size was calculated using Cochran’s formula, assuming a 5% level of significance and an estimated rotavirus prevalence of 80% among children from previous studies. The minimum sample size was calculated as 216. To account for possible data incompleteness or loss, the final target sample size was adjusted to 226. Participants were selected through convenience sampling based on daily admissions that met the inclusion criteria.

### Data collection and study procedure

After obtaining written informed consent from the parents or legal guardians, a structured questionnaire was administered to collect data on sociodemographic characteristics, feeding practices, vaccination history, clinical symptoms, nutritional status, and environmental factors. Clinical examination findings, including signs of dehydration and fever, were recorded by trained medical personnel.

Stool samples were collected from each participant within 24 hours of admission and tested for rotavirus antigen using enzyme-linked immunosorbent assay techniques following standard laboratory protocols.

### Variables and definitions

• **EBF:** Defined as feeding with only breast milk, without any additional food or drink (including water), for the first 6 months of life.
• **Feeding status:** Classified as EBF, non-EBF (partial or no breastfeeding), and mixed/complementary feeding.
• **Rotavirus positivity:** Defined by the presence of rotavirus antigen in stool samples.
• **Nutritional status:** Assessed by mid–upper arm circumference (MUAC) and categorized according to WHO guidelines into severe acute malnutrition (SAM), moderate acute malnutrition (MAM), and well-nourished.

### Ethical considerations

The study protocol was reviewed and approved by the Institutional Review Board of Chittagong Medical College. Written informed consent was obtained from the parents or guardians of all enrolled participants. Data confidentiality and anonymity were strictly maintained throughout the study.

### Data analysis

Data were analyzed using Stata 14.0 (StataCorp. 2015. Stata Statistical Software: Release 14. College Station, TX: StataCrop LP).

**Descriptive analysis:**

○ Descriptive statistics were used to summarize sociodemographic, clinical, nutritional, and feeding characteristics of the study population.
○ Categorical variables (e.g., sex, residence, breastfeeding status) were summarized using frequencies and percentages.
○ Continuous variables (e.g., duration of diarrhea, number of motions per day) were described using means and standard deviations (SD).

**Rotavirus status analysis:**

○ The proportion of children who tested positive for rotavirus was calculated.
○ Sociodemographic and feeding practices were compared between rotavirus-positive (RV+) and rotavirus-negative (RV–) groups using frequency tables.

**Bivariate analysis:**

○ Chi-square tests (χ²) were used to examine associations between rotavirus positivity and categorical variables such as:
  ■ EBF
  ■ Dehydration status
  ■ Appearance and thirst status
  ■ Vaccination history
○ Fisher’s exact test was used when cell counts were fewer than 5.

**Odds ratio estimation**

○ Unadjusted odds ratios (OR) with 95% confidence intervals (CIs) were calculated to estimate the strength of association between feeding practices and rotavirus infection, particularly among:
  ■ Children ≤6 months
  ■ Children >6 months
○ Reference groups were defined for each comparison (e.g., EBF as the reference category).

**Severity of diarrhea analysis:**

○ Severity of diarrhea was categorized (mild, moderate, severe, maximum score) using the Vesikari scale.
○ The association between diarrhea severity and feeding practices was assessed using cross-tabulations and χ² tests.
○ Separate analyses were performed for children ≤6 months and >6 months.

**Nutritional status assessment:**

○ MUAC was used to categorize nutritional status into SAM, MAM, and well-nourished groups.
○ MUAC status was compared with RV+ descriptively.

**Statistical significance:**

A *P*-value <.05 was considered statistically significant for all inferential tests.

## Results

A total of 226 participants were enrolled among 1234 children under 2 years of age hospitalized with acute watery diarrhea during the study period. The majority (64%) belonged to the 7–12-month age group, followed by 25% in the 13–18-month group. Equal distribution was observed between male and female participants. Among parents, 38% of fathers and 48% of mothers had completed secondary-level education. The majority of mothers (86%) were housewives, and 58% of the families reported monthly household expenditures of ≤10,000 BDT. About half (50%) of the participants resided in rural areas, and 41% lived in pakka houses. Tube wells (48%) and municipal supply water (40%) were the primary sources of drinking water, while sanitary latrines were used in 84% of households (Table 1).

**Table 1:** Demographic and socioeconomic characteristics of hospitalized children (N = 226).

Demographic and socioeconomic characteristics	Category	n (%)
**Age group (months)**	≤6	18 (8)
	7-12	145 (64)
	13-18	56 (25)
	19-23	7 (3)
**Sex**	Male	114 (50)
	Female	112 (50)
**Father’s education**	Illiterate	50 (22)
	Primary	54 (24)
	Secondary	85 (38)
	≥HSC	37 (16)
**Mother’s education**	Illiterate	46 (20)
	Primary	51 (23)
	Secondary	109 (48)
	≥HSC	20 (9)
**Father’s occupation**	Day labor	54 (24)
	Farmer	7 (3)
	Business	34 (15)
	Service	58 (26)
	Others	73 (32)
**Mother’s occupation**	Housewife	194 (86)
	Working	32 (14)
**Monthly expenditure**	≤10,000	131 (58)
	10,001–20,000	77 (34)
	>20,000	18 (8)
**Residence**	Rural	113 (50)
	Semi-urban	40 (18)
	Urban	43 (19)
	Slum	30 (13)
**House type**^a^	Kacha	48 (21)
	Semi-kacha	34 (15)
	Pakka	91 (41)
	Tin	25 (11)
	Others	28 (12)
**Water source**	Tube well	110 (48)
	Supply	90 (40)
	Others	26 (12)
**Latrine type**	Sanitary	193 (84)
	Others (unimproved)	33 (16)

a Kacha = mud/temporary, Semi-kacha = partially permanent, Pakka = permanent, Tin = tin-roofed, Others = not categorized above.

Among all 226 children hospitalized with AWD, the majority were aged 7-12 months (64%), with equal sex distribution. EBF was more common among children ≤6 months. Severe diarrhea occurred in 60% of children, and most presented with increased thirst and irritability. Nutritional status was variable, with 16% classified as severely malnourished. No significant differences in severity were observed by feeding practices, though a trend toward less severe diarrhea among EBF children was noted.

Of the total 226 children, 60% (n = 135) tested positive for rotavirus (RV+). Feeding practices among 135 RV+ children showed that 98.5% had a history of breastfeeding, but 34.8% were given other milk, 79.3% had consumed solid food, and 31.1% were bottle-fed. Mixed feeding was reported in 43% of cases, while 27.4% were predominantly milk-fed (Table 2).

**Table 2:** Feeding practices and rotavirus positivity (N = 135 rotavirus + cases).

Feeding practice	Category	n (%)
**Breastfed ever**	Yes	133 (98.5)
	No	2 (1.5)
**Other milk intake**	Yes	47 (34.8)
	No	88 (65.2)
**Solid food intake**	Yes	107 (79.3)
	No	28 (20.7)
**Bottle feeding**	Yes	42 (31.1)
	No	93 (68.9)
**Predominant food**	Milk	37 (27.4)
	Complementary	8 (5.9)
	Mixed	58 (43)
	Others	32 (23.7)

In both age groups (≤6 months and >6 months), not being exclusively breastfed significantly increased the risk of rotavirus infection. For children aged ≤6 months, the odds of rotavirus positivity were 10 times higher in non-EBF children compared with EBF (OR: 10.0, 95% CI: 4.81-17.97; *P* =.04). For those aged >6 months, the odds were 9.72 times higher (OR: 9.72, 95% CI: 4.51-20.94; *P* <.001) (Table 3).

**Table 3:** Association between EBF and rotavirus positivity.

Age group	Feeding practice	% Rotavirus+	Odds ratio (95% confidence interval)	*P*-value
**≤6 months** **(n = 18)**	EBF	66.6%	Reference	–
	Not EBF	16.6%	10.0 (4.81–17.97)	0.04
**>6 months** **(n = 208)**	EBF	38.9%	Reference	–
	Not EBF	86.1%	9.72 (4.51–20.94)	<0.001

EBF, exclusive breastfeeding.

Clinical presentation showed that vomiting was significantly more common in RV+ children (57.8% vs 42.9%, *P* =.031). Other clinical features—including fever, dehydration, appearance, thirst, nutritional status, and diarrhea severity—showed no significant differences between RV+ and RV– children. Severe diarrhea was more frequent in RV+ cases (65.2% vs 52.7%), but this was not statistically significant. Mean diarrhea duration and maximum motions per day were slightly higher in RV+ children, while admission temperatures were comparable (Table 4).

**Table 4:** Clinical characteristics, nutritional & vaccine status of rotavirus positive and negative hospitalized children (N = 226).

Clinical feature	Rota+ (n = 135)	Rota– (n = 91)	Total (N = 226)	χ² (df)	*P*-value
**Vomiting**	78 (57.8)	39 (42.9)	117 (52.0)	4.65 (1)	0.031
**Fever**	64 (47.4)	34 (37.4)	98 (43.4)	2.14 (1)	0.144
**Pre-hospital medication**	72 (53.3)	46 (50.5)	118 (52.2)	0.14 (1)	0.707
**Pre-hospital IV fluid**	7 (5.2)	7 (7.7)	14 (6.2)	0.61 (1)	0.433
**Diarrhea severity**	**Mild:** 45 (33.3) **Moderate:** 2 (1.5) **Severe:** 88 (65.2)	38 (41.8) 1 (1.1) 48 (52.7)	83 (36.7) 3 (1.3) 136 (60.2)	3.56 (2)	0.168
**Vaccine coverage**	1 (0.7)	3 (3.3)	4 (1.8)	2.16 (1)	0.142
**Dehydration**	**None:** 59 (43.7) **Some:** 75 (55.6) **Severe:** 1 (0.7)	43 (47.3) 44 (48.4) 4 (4.4)	102 (45.1) 119 (52.7) 5 (2.2)	3.97 (2)	0.137
**Appearance**	**Normal:** 19 (14.1) **Irritable:** 109 (80.7) **Lethargic:** 7 (5.2)	11 (12.1) 73 (80.2) 7 (7.7)	30 (13.3) 182 (80.5) 14 (6.2)	0.72 (2)	0.699
**Thirst**	**Normal:** 24 (17.8) **Increased:** 111 (82.2)	13 (14.3) 78 (85.7)	37 (16.4) 189 (83.6)	0.48 (1)	0.487
**MUAC status**	**SAM:** 26 (19.3) **MAM:** 55 (40.7) **AM:** 29 (21.5) **Normal:** 25 (18.5)	11 (12.1) 35 (38.5) 22 (24.2) 23 (25.3)	37 (16.4) 90 (39.8) 51 (22.6) 48 (21.2)	3.14 (3)	0.371
**Mean diarrhea duration (days, mean ± SD)**	2.6 ± 1.1	2.4 ± 1.0	2.53 ± 1.09	—	—
**Max motions/day (mean ± SD)**	12.2 ± 9.5	11.2 ± 9.0	11.84 ± 9.38	—	—
**Temperature on Admission**	**Hypothermia:** 45 (33.3) **Normal:** 70 (51.9) **Hyperthermia:** 20 (14.8)	24 (26.4) 52 (57.1) 15 (16.5)	69 (30.5) 122 (54.0) 35 (15.5)	1.12 (2)	0.571

MUAC, mid upper arm circumference; AM, acute malnutrition; MAM, moderate acute malnutrition; SAM, severe acute malnutrition.

Although not statistically significant, clinical signs such as some dehydration (55.6%) and increased thirst (82.2%) were more prevalent among RV+ children compared with negative cases. In terms of appearance, most children in both groups were irritable (Table 5).

**Table 5:** Clinical signs and association with RV positivity

Variable	Category	RV+ n (%)	RV– n (%)	*P*-value
**Dehydration**	None	59 (43.7)	43 (47.3)	0.137
	Some	75 (55.6)	44 (48.4)	
	Severe	1 (0.7)	4 (4.4)	
**Appearance**	Normal	19 (14.1)	11 (12.1)	0.699
	Irritable	109 (80.7)	73 (80.2)	
	Lethargic	7 (5.2)	7 (7.7)	
**Thirst**	Normal	24 (17.8)	13 (14.3)	0.487
	Increased	111 (82.2)	78 (85.7)	

RV, rotavirus.

Among children ≤6 months, those who were EBF tended to have a higher proportion of mild diarrhea (57.8%) compared with non-EBF (28.9%) or mixed-fed children (13.3%). For children >6 months, mixed/other feeding was most common and contributed to a higher number of severe cases (64.0%). Overall, although there was a trend toward less severe diarrhea among EBF children, the differences were not statistically significant (*P* =.52 for ≤6 months, *P* =.18 for >6 months) (Table 6).

**Table 6:** Association between feeding practice and severity of diarrhea among hospitalized children (N = 226).

Age group	Feeding practice	Mild n (%)	Moderate n (%)	Severe n (%)	χ²	*P*-value
**≤6 months**	EBF	48 (57.8)	2 (66.7)	71 (52.2)	5.2	0.52
	Not EBF	24 (28.9)	1 (33.3)	50 (36.8)		
	Mixed/Others	11 (13.3)	0 (0)	15 (11.0)		
**>6 months**	EBF	5 (6.0)	0 (0)	24 (17.6)	8.9	0.18
	Not EBF	19 (22.9)	0 (0)	25 (18.4)		
	Mixed/Others	59 (71.1)	3 (100)	87 (64.0)		

EBF, exclusive breastfeeding.

## Discussion

This study revealed a 60% rotavirus positivity (RV+) rate among children under 2 years of age hospitalized with AWD, which is notably higher than the 40% rotavirus detection reported among children under 5 years of age in a 2012 hospital-based study in Bangladesh [16]. The increased detection in our study could be attributed to the younger age group, as rotavirus infection is known to be more prevalent in children under 2 years [8,10]. Similar trends were reported in a Myanmar hospital-based study, where rotavirus was identified in 37% of hospitalized diarrhea cases [17].

Our findings align with studies from India and New Zealand that highlight a peak incidence between 6 and 23 months [18,19]. In our study, children aged 7-12 months had the highest rotavirus positivity (63%). Krawczyk et al. [18] and Prameela and Vijaya et al. [19] reported similar age-specific trends in Kerala and New Zealand, respectively. Gender distribution was equal in our cohort, consistent with findings from Kosovo [20] and national demographic data [13,21,22].

The majority of infected children presented with vomiting and fever, consistent with symptoms reported by studies in Myanmar and India [17,23]. Regarding dehydration, 56% of RV+ children had some dehydration, and 1% had severe dehydration, which is similar to Myanmar findings [17].

This study highlighted the low rotavirus vaccine coverage (1.8%), underscoring an urgent need to improve immunization uptake [11]. Nutritional status remains a concern, as more than 55% of children were acutely malnourished, echoing findings from Mozambique and Zambia linking rotavirus vulnerability to undernutrition [24].

The protective role of EBF was supported in our study. The odds of rotavirus infection were significantly higher among children not exclusively breastfed, both in the ≤6-month and >6-month age groups. A meta-analysis found EBF to be protective in 83% of studies compared to partial or no breastfeeding [25]. Similar results were found in Uganda, India, and Sri Lanka [17,18,26].

Furthermore, breastfed children tend to experience milder symptoms, as reflected in our study, where exclusively breastfed children were less likely to have severe dehydration or high diarrhea scores. This supports findings that breast milk contains immunologic factors that help protect against gastrointestinal pathogens [20,23,27].

This study is subject to some limitations. First, data on EBF were based on parental recall, which may introduce recall bias. Second, the study was conducted in a single tertiary hospital and included only hospitalized children, which may not reflect community-level patterns and introduces selection bias. Finally, as participants were enrolled through convenience sampling, the findings may not be fully generalizable to the wider pediatric population of Bangladesh.

## Conclusion

Almost 60% of the children under 2 years of age hospitalized with AWD were infected with rotavirus. The infection was more common among children aged 6-12 months. Fewer than 2% of children had received the rotavirus vaccine. A significant proportion of RV+ cases (64%) lacked a history of EBF. The odds of rotavirus positivity were approximately 10 times higher in non-exclusively breastfed children compared to exclusively breastfed peers. These findings support the protective effect of EBF against rotavirus infection.

## Recommendations

To reduce the burden of rotavirus-related diarrhea in children under 2 years of age, authorities should:

1. Promote EBF for the first 6 months of life.
2. Encourage continued breastfeeding until 2 years of age.
3. Strengthen rotavirus vaccination programs.
4. Conduct large-scale case-control studies for further insights.

## Declaration of competing interest

The authors have no competing interests to declare.
